# The complete plastome of *Daphne laureola* L. (Thymelaeaceae)

**DOI:** 10.1080/23802359.2019.1674201

**Published:** 2019-10-04

**Authors:** Kálmán Könyves, Sawita Yooprasert, Alastair Culham, John David

**Affiliations:** a Royal Horticultural Society Garden Wisley, Woking, UK;; b Herbarium, School of Biological Sciences, University of Reading, Whiteknights, UK

**Keywords:** Daphne, Thymelaeaceae, plastome, SSC

## Abstract

The complete plastome sequence for the type species of *Daphne*, *Daphne laureola*, was assembled and annotated in this study. The total length of the *D. laureola* plastome is 171,613 bp and comprises the large single copy (LSC) spanning 85,316 bp, the small single copy (SSC) spanning 2855 bp, and two inverted repeat (IR) regions each of 41,721 bp length. The SSC contains only two genes: *ndhF* and *rpl32*. This sequence extends the list of Thymelaeaceae plastomes to be used in future systematic studies of the family and is the first European species to be sampled.

*Daphne* is a genus of small shrubs, popular with gardeners for their fragrant flowers. The systematics of *Daphne*, and its relationship to *Wikstroemia*, is poorly understood (Wang et al. 2007). *Daphne laureola* L. is an evergreen shrub native to Europe and North Africa and is the type species of the genus. Establishing the level of genetic divergence within a phylogenetic framework will help understand taxonomic boundaries within the genus and between related genera. Here we report the complete plastome sequence of *D. laureola.*

Leaf material was collected in silica gel from *D. laureola* grown at RHS Garden Wisley, UK (51.312695° N, 0.476724° W). The sample was further verified as *D. laureola* by a BLAST search using the 3’-end of *ndhF*. A herbarium voucher was deposited at **WSY** (WSY0148635). Total genomic DNA was extracted using a CTAB protocol (Doyle and Doyle 1987). Library development and 150 bp PE sequencing using Illumina HiSeq were done by Novogene Corporation (Beijing, China). Raw sequence data were deposited in SRA (accession PRJNA555571). The plastome was assembled with Fast-Plast v1.2.6 (McKain and Wilson 2017) and NovoPlasty v2.7.0 (Dierckxsens et al. 2017). Fast-Plast assemblies were run with all 29.4 M reads. Reads were trimmed to remove NEB-PE adapter sequences. The Bowtie reference index was built with Malvales plastomes included in Fast-Plast. For the NovoPlasty assembly, a *matK* sequence of *D. laureola* (JN894952) was used as starting seed and memory was limited to 8 Gb. All other parameters were unchanged. The large single copy (LSC), the small single copy (SSC), and two inverted repeat (IR) regions were identified in the final Fast-Plast contig and NovoPlasty assemblies and the circular plastome was assembled by hand using Geneious v11.1.5 (http://www.geneious.com, Kearse et al. 2012). Junctions of the single copy and inverted repeat regions were confirmed by mapping the reads to the ends of the IR and identifying the point where the mapped reads diverged.

The mean coverage of the finished assembly is 521×. The complete plastome was annotated by transferring the annotations from *D. kiusiana* (KY991380) using Geneious v11.1.5 and corrected by comparing it with other published annotations. The *D. laureola* plastome sequence was aligned to 13 published Malvales plastome sequences plus *Arabidopsis thaliana* (outgroup) using MAFFT v7.388 (Katoh and Standley 2013). A maximum likelihood estimate of phylogeny was conducted with RAxML v8.2.11 (Stamatakis 2014) within Geneious v11.1.5 using model GTR + G and 1000 bootstrap replicates.

The plastome sequence of *D. laureola* (MN201546) is 171,613 bp, comprises the LSC spanning 85,316 bp, the SSC spanning 2,855 bp, and two IR regions each of 41,721 bp length. The IRs have expanded to encompass most of the genes normally in the SSC, which contained only two genes *ndhF* and *rpl32*. This structure is congruent with the published *Daphne* plastomes. The Thymelaeaceae samples form a clade with *D. laureola* sister to the remaining *Daphne* species (Figure 1).

**Figure 1.**
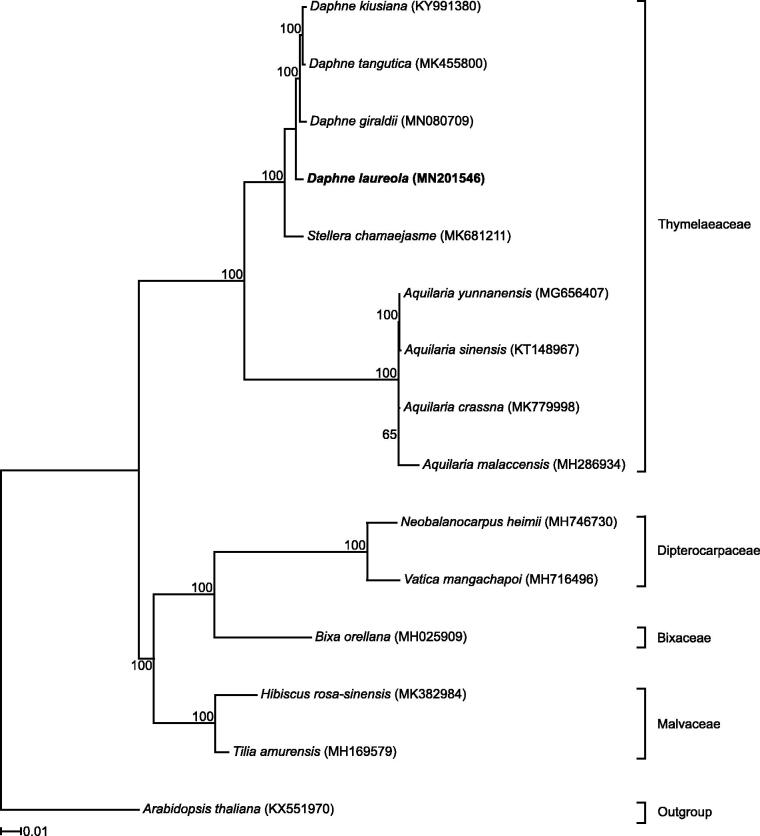
RAxML output tree with bootstrap consensus values based on 15 complete plastome sequences. The numbers at each node indicate bootstrap support. GenBank accession numbers are given in brackets. Text in bold shows the plastome developed in this study. Families of the sampled taxa are shown on the right.
